# Formative research to develop a community-based intervention for chronic disease prevention in Guatemalan school-age children

**DOI:** 10.1186/1471-2458-14-101

**Published:** 2014-01-31

**Authors:** Paola Letona, Manuel Ramirez-Zea, Benjamin Caballero, Joel Gittelsohn

**Affiliations:** 1 INCAP Comprehensive Center for the Prevention of Chronic Diseases, Institute of Nutrition of Central America and Panama, Guatemala City 1118, Guatemala; 2 Department of International Health, Center for Human Nutrition, Johns Hopkins Bloomberg School of Public Health, Baltimore, MD 21205, USA

**Keywords:** Formative research, Community-based intervention, Behavioral risk factors, Chronic disease prevention, Developing countries

## Abstract

**Background:**

Noncommunicable diseases (NCD) are the most common causes of morbidity and mortality worldwide, even in low- and middle-income countries (LMIC). Recent trends in health promotion emphasize community-based interventions as an important strategy for improving health outcomes. The aim of this study was to conduct formative research regarding the perceptions of NCD risk factors, their influencing factors, and community resources available to aid the development and implementation of a community-based intervention with school-age children.

**Methods:**

Focus group discussions (n = 18), home visits (n = 30), and individual semi-structured interviews (n = 26) were conducted in three urban communities in Guatemala with school-age children (10–12 years of age), teachers, parents, and local community members (i.e., school principals, school food kiosk vendors, religious leaders, authority representatives). All focus groups and interviews were transcribed verbatim for thematic analysis.

**Results:**

Children, parents, and teachers have general knowledge about modifiable risk factors. Adults worried more about tobacco use, as compared to unhealthy diet and physical inactivity in children. Participants identified features at the intrapersonal (e.g., negative emotional state), interpersonal (e.g., peers as role models), and organizational and community levels (e.g., high levels of crime) that influence these risk factors in children. School committees, religious leaders, and government programs and activities were among the positive community resources identified.

**Conclusions:**

These findings should help researchers in Guatemala and similar LMIC to develop community-based interventions for NCD prevention in school-age children that are effective, feasible, and culturally acceptable.

## Background

Noncommunicable diseases (NCD) are the most common causes of morbidity and mortality worldwide. In 2008, 36 million deaths (63% of total deaths) were due to NCD, mainly cardiovascular diseases, diabetes, and cancer [1]. Approximately 80% of NCD deaths occur in low- and middle-income countries (LMIC), and 25% of deaths are in individuals younger than 60 years of age. The World Health Organization projects that, globally, NCD deaths will increase by 15% between 2010 and 2020 [2].

Most NCD can be prevented by reducing four behavioral risk factors: unhealthy diet (i.e., high saturated fat and sugar intake, low fruit and vegetable intake), physical inactivity, tobacco use, and alcohol consumption [3]. While NCD are apparent in adulthood, these behaviors usually start in childhood and adolescence and have proven difficult to change. Therefore, the development and implementation of community-based interventions in children of LMIC, who are particularly vulnerable to developing NCD as adults, may be a way to positively influence behavior before unhealthy habits are formed [4].

Recent trends in health promotion emphasize community-based interventions as an important strategy to improve health outcomes. However, evidence of their effectiveness varies. While there is evidence for increased physical activity [5] and fruit and vegetable intake [6,7], there is unclear evidence for the prevention of smoking [8] and alcohol consumption [9]. Modest impact of community-based interventions has been attributed to secular trends, barriers of the intervention, theoretical framework, and insufficient formative research [10]. Hence, formative research is needed to develop effective interventions that are culturally and geographically appropriate [11].

In Guatemala, a LMIC, it is estimated that NCD account for 47% of all deaths [12]. However, little is known about the prevalence of NCD risk factors in school-age children. In the capital of the Department of Quetzaltenango, the second largest city in Guatemala, it was found that 56% of the children attending third and fourth grade had inadequate fruit and vegetable intake [13], less than 400 g a day [14]. Smoking prevalence in adolescents and young men (aged 15–24 years) has been reported at 23% [15]. In a study conducted in the capital of the Department of Chimaltenango, 43% of the children aged 8–13 years had high triglycerides, 17% had low high-density lipoprotein (HDL) levels, and 12% were obese [16].

As of January 2014, Guatemala lacks community interventions that address NCD risk factors in children. In order to develop effective intervention strategies, formative research studies are needed to assess community attributes and the context in which the development of NCD risk factors may occur during childhood, as well as to suggest appropriate messages, communications channels, and promotional strategies.

In this paper, we describe the procedures and results of a formative research study that was used to aid the development and implementation of a community-based intervention designed to prevent NCD risk factors in children. The study was guided by the ecological model for health promotion that focuses on intervening intrapersonal, interpersonal, organizational, community, and public policy factors that encourage and sustain unhealthy behaviors [17]. Data were collected from all the levels (except public policy) that can be changed through a community-based intervention.

Our primary goal was to collect information that could be used to develop a culturally appropriate community-based intervention to prevent NCD later in life. We focused on answering these questions: What are the perceptions and concerns about risk factors for NCD in Guatemalan school-age children? What factors at the intrapersonal, interpersonal, organizational, and community levels influence risk factors for NCD? What community resources are available for promoting healthy lifestyles in children?

## Methods

Qualitative research was conducted, and included focus group discussions (FGD) with school-age children (4^th^ – 6^th^ Grade), parents, and teachers; home visits to parents; and individual semi-structured interviews with local community members (i.e., school principals, school food kiosk vendors, religious leaders, authority representatives) (Table 1).

**Table 1:** Formative research data collection methods

**Method**	**Types of participants**	**No. conducted (no. of participants)**
Focus group discussions	Children	6 (32)
	Parents	6 (34)
	Teachers	6 (29)
Home visits	Parents	30
Semi-structured interviews	School principals	6
	School food kiosk vendors	6
	Religious leaders	5
	Authority representatives	9

### Setting

The study was conducted from June to September 2010 in the Department of Guatemala. Three low-income urban communities (i.e., Mixco, Santa Catarina Pinula, Villa Nueva) were identified and six low-income elementary schools, four public and two private, were selected based on convenience; two schools from each community. Previous publications have used type of school (public or private) to classify children in public schools as low socio-economic status, and those of private schools (monthly fees US$60-$120) as high socio-economic status [13,18]. However, in this study, children attending private schools (monthly fee < US$16) were still considered as being of low socio-economic status.

The Institutional Ethics Committee of the Institute of Nutrition of Central America and Panama (INCAP) in Guatemala City, and the Institutional Review Board of the Johns Hopkins School of Public Health approved the study protocol and informed consent.

### Participants

Permission to work at the schools was obtained from the School District Supervisors and school principals. Fourth to sixth grade teachers were informed of the study and invited to participate in a FGD. The school principals provided the lists of students in the fourth to sixth grades (aged 10–12 years), and children’s names were randomly selected for FGD with children, FGD with parents, and for home visits. Parents from the selected children were invited to a school meeting where a member of the research team explained the study procedures, voluntary participation, confidentiality, and consent form. Only children with parental consent and child oral assent were recruited.

In addition, school principals, school food kiosk vendors, popular Catholic and Protestant religious leaders, and authority representatives (i.e., Ministry of Education, Municipality, Health Center) that may directly or indirectly influence children’s environment at the school or elsewhere in their community were purposively selected for semi-structured interviews.

### Data collection

#### FGD

A total of 18 FGD were conducted, three in each school: one with children (alternated boy and girl FGD between schools), one with parents (who had a child in fourth, fifth, or sixth grade), and one with teachers. The discussion guide used with all the groups was similar and focused in exploring their knowledge and attitudes towards sugar-sweetened beverages, energy dense snack foods (e.g., potato chips), fruits and vegetables, physical activity, and tobacco; and perceptions of factors influencing high saturated fat and sugar intake, low fruit and vegetable intake, physical inactivity, and tobacco use in children aged 10 – 12 years old. All FGD were conducted in an empty classroom at each school. The discussions lasted an average of 28 minutes with children, 62 minutes with parents, and 48 minutes with teachers.

#### Home visits

A total of 30 home visits (five per school) were conducted. Each home visit combined an individual interview with a parent (who had a child in the fourth, fifth, or sixth grade) and an unstructured observation (to permit exploration) to assess characteristics of the home environment related to physical activity. In 93% (n = 28) of the home visits the mother was interviewed, and in the other two households a father and a grandmother participated. The semi-structured interview guide covered family topics such as food preparation, weekday and weekend routine, entertainment activities, and opportunities to do physical activity. Permission was asked to observe the areas where the family watches television (if they had one), and where their children usually play actively. A form was filled out with the summarized answers of the parents and the details of the areas observed at the house. The home visits lasted an average of 35 minutes (range 30 to 45).

#### Individual semi-structured interviews

A total of 26 local community members (i.e., school principals, school food kiosk vendors, religious leaders, authority representatives) were interviewed. The questions in the interview guides were different for each type of community member, but focused on identifying resources related to the social and physical environment (e.g., people, places, programs, organizations) that could strengthen a community-based intervention aimed to prevent the development of NCD risk factors in school-age children. School principals were asked about school organization, responsibilities of its personnel, and institutions that have benefited their school and students; food vendors about their working experience (e.g., economic situation, most popular food, attempts of selling healthier food); religious leaders about the activities they carry out that promote healthy lifestyles; and authority representatives about successful community programs that promote healthy lifestyles in children and their families.

### Data analysis

All FGD and individual semi-structured interviews were audio recorded and transcribed verbatim in Spanish. Information obtained from the home visits was transferred into an electronic document. Transcripts were imported to ATLAS.ti (version 6.2) [19] for coding, organization, and thematic analysis. Specific codes were organized into a series of themes that addressed the three primary research questions of the study. Analysis was conducted in Spanish by PL (a bilingual Guatemalan researcher), who translated into English those excerpts selected for quotations.

Methodological triangulation was used in the analyses, comparing and contrasting information from each of the different forms of data collection, from the three groups, children, parents, and teachers.

## Results

### Perceptions about risk factors for NCD

#### Energy dense snack foods

All informants (i.e., children, parents, teachers) reported that energy dense snack foods (i.e., chips, candy, sugar-sweetened beverages, soft drinks) are unhealthy. Children explained that they *“harm our body”, “damage teeth”, “don´t have vitamins”,* and *“give diseases”,* whereas parents and teachers mentioned that they have *“chemicals”, “a lot of fat”,* and *“a lot of calories”.* All adults agreed that children like the taste and eat them frequently.

*“…my kids…they really enjoy chips, they cannot have a Quetzal* [currency of Guatemala] *in their hand because they will soon go buy chips, the four of them love chips…”* (Mother)

#### Fruits and vegetables

Fruits and vegetables are generally perceived as being healthy and good for the body because they contain vitamins. The majority of children expressed that they like fruits but were more selective when they talked about vegetables, often listing a maximum of four vegetables that they like compared to an indefinite number of fruits. Salads (often prepared with greens and/or raw vegetables) were mentioned as one of the most common mix of vegetables liked by children.

*“Sometimes you don’t feel like eating vegetables. At times, I do feel like it. Sometimes I go and buy a bunch of radishes, three cucumbers, and I make a salad for me. I like it that way.”* (Boy)

Many parents talked about their children’s rejection of vegetables and expressed that it is common to see their children taking out the vegetables from the meals they prepare (e.g., mixed rice, chicken and vegetables, soup). Some parents expressed confusion about why their children started rejecting vegetables between the ages of seven and nine, even though they used to eat them at an early age.

#### Physical activity

Physical activity in children mostly occurs through active play during and after school. Children mentioned playing ball, soccer, or jumping rope as their favorite activity to do when they have free time. Although, all the informants knew that physical activity is a healthy habit, they did not have knowledge about the recommended levels of physical activity for children. Children were the only ones that mentioned some physical and social benefits of physical activity: *“keeps the body in shape”* and *“away from bad influences.”*

During home visits (n = 30), most parents mentioned that television viewing is a common family activity during weekdays and weekends. Their only concern was that children might be exposed to violence, sex, and aggression. It was observed that 100% of the households had at least one television.

#### Tobacco

All informants had more knowledge about the consequences of tobacco use than any other modifiable risk factor considered. Children and parents talked about lung damage, lung cancer, respiratory problems, cardiovascular disease, and addiction. Second hand smoke was rarely mentioned.

*“…I talk to the children about active and passive smoking, which for a long time I did not know about. It was only until recently that I heard that being around someone who smokes can affect my health.”* (Teacher)

Parents and teachers perceived that low vegetable intake and high saturated fat and sugar intake are very common in children, but expressed that they are most concerned about tobacco use, since they consider it a behavior they cannot always control in children.

*“Overweight, I can control at home, but tobacco is my concern, but in that case only God* [can control it].*”* (Mother)

Although alcohol consumption in children was not explored, parents and teachers often mentioned it when they talked about tobacco. They stressed the importance of preventing its consumption because it is a behavior they have seen in children of this age.

### Factors that influence NCD risk factors in children

**Figure 1:**
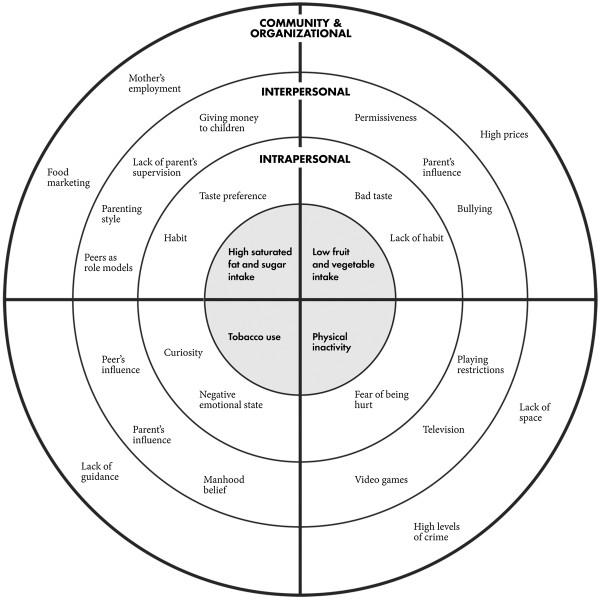
Ecological model of the factors that influence NCD risk factors in Guatemalan children.

The most salient factors that children, parents, and teachers mentioned that influence high saturated fat and sugar intake, low fruit and vegetable intake, physical inactivity, and tobacco use in school-age children are shown in Figure 1. These factors are organized into the different levels of the ecological framework: intrapersonal, interpersonal, and organizational and community. Organizational and community levels where grouped together because several factors were found to simultaneously influence both levels (i.e., high prices, lack of space, lack of guidance, food marketing). In the text that follows, each factor is described within the context of each behavior to better understand the interaction among different levels.

#### High saturated fat and sugar intake

Most children prefer energy dense snack foods because they taste good and are used to eating them. Parents and teachers explained that often eating snack foods could turn into a bad habit for children.

Many informants expressed that mothers seem to have a strong influence in their children’s eating habits, both positive and negative. Many mothers seek employment to help with their family income. As a result, some lack the time and energy to prepare school snacks and meals and prefer to give money to their children; hence, mothers no longer know what their children eat at school. In addition, some mothers are permissive and often agree to buy their children snack foods as a reward for good behavior, when they don’t want them to cry, or because they are unable to establish limits and say *“no.”*

*“In order for children to eat their vegetables, some parents tell them: ‘if you eat your vegetables I am going to give you cookies.’”* (Girl)

Children also are influenced by the opinions and snack choices of their more popular peers. Sometimes they reject foods because they hear others saying it tasted bad or ask their mothers for money so they can buy the same food as their peers.

All informants recognized the strong influence that food marketing has on children’s low cost snack choices. Although children participating in the FGD expressed that food marketing strategies are more effective with younger children (1^st^ – 3^rd^ Grade); parents and teachers agreed that it influences children of all ages. The most mentioned food marketing strategies were food packaging (e.g., attractive colors and designs), collectibles (e.g., stickers, toys), and snack promotions.

#### Low fruit and vegetable intake

Most children expressed liking fruits but not vegetables. They argued that vegetables *“feel odd”, “lack flavor”,* or *“taste bitter”.* Some mothers shared that their children have tantrums when served vegetables, and that mothers often prevent a tantrum by allowing their children not to eat their vegetables.

*“…when he* [father] *is present, they* [children] *have to eat everything…when he is not there, I prefer to allow it ‘ok, don’t eat it then.’”* (Mother)

Many informants expressed that the opinion and food preferences of parents may influence children’s vegetable intake. For example, they said that some fathers express their dislike for vegetables in front of their children or ask their wife not to prepare them at home.

At school, some boys (mostly sixth graders) mentioned that they are bullied or mocked for eating vegetables, and are often called *“worm”, “momma’s boy”, “poor”,* or *“vegetarian”.* As a result, they ask their mothers not to send them to school with vegetables.

The majority of informants considered the price of fruits and vegetables to be high, but the teachers were the ones who expressed that this affects the children’s possibility to eat them at home or school. They also argued that the school food kiosk often sells snacks that are cheaper than fruits and vegetables.

*“I had some students that said to me when I taught nutrition…‘Miss, we eat vegetables once every eight days and meat once a month when my dad gets paid.’”* (Teacher)

#### Physical inactivity

Many children expressed that their playtime is restricted both at school and at home. Teachers explained that some schools are overcrowded and need more space for students to play safely during recess. As a result, teachers often scold children for playing actively and some children prefer not to play because they fear getting hurt. The majority of parents expressed that they do not give their children permission to play outside the house because of the high levels of crime in their neighborhoods (e.g., robberies, violence, shootings, murders, youth gangs), and lack of safe places for children to play. During the home visits, it was observed that only 63% of the houses (n = 19) had at least one small space inside it where children can play (e.g., patio, garage, corridor).

“…*the parents prefer to have him* [child] *locked inside the house, than to let the child go out to the street to find a gang member or find other bad influences. It is better that he* [child] *stays inside the house…”* (Teacher)

All parents interviewed during the home visits mentioned that watching television was one of their main entertainment activities as a family. Parents are not accustomed to setting television limits. Many children expressed that they can watch television without time constraints after finishing their homework. Even though video games (i.e., console, computer, cell phone) were mentioned as a factor that might be influencing physical inactivity, only 10% (n = 3) of the households had a video game console.

#### Tobacco use

Peers and parents who smoke were considered the strongest influence for smoking initiation in children. On the one hand, most informants recognized that many children start smoking out of curiosity. Children expressed that when they see others smoke, they want to know how it feels and tastes. On the other hand, peers and adults (adult men) often pressure children to adopt this behavior. There is a belief that smoking proves the boy’s manhood.

*“Sometimes they* [other children and adult men] *say that because I am a man I have to do it* [smoke], *but that is not true.”* (Boy)

In addition, negative emotional states (e.g., sadness, despair, disappointment, feeling unloved) that arise from family problems were also seen as possible contributors to smoking initiation in children.

Parents perceive that there is lack of guidance about tobacco at schools, and teachers perceive that the guidance is missing at home; therefore, they consider it important for teachers and parents to talk about the consequences of smoking in order to prevent it.

### Potential strategies for promoting children’s healthy lifestyles

Several community resources/strategies were identified of relevance for the implementation of an intervention, each with its own barriers.

#### Work with school committees

Public school principals noted that they have several committees (e.g., finance, cleaning, sports, culture, snack) formed by teachers and some parents that help support and carry out administrative and extracurricular activities. However, their performance is often impaired by the lack of communication teachers and parents have with the school principal.

*“Everyone of us has a technical field and desire to work…but the ideas and desire to work end here* [school], *because everything is cut* [by the school principal].*”* (Teacher)

*“…if the school principal would let us, I think we would be very effective.”* (Teacher)

#### Provide educational resources to teachers

The National Curriculum of Education includes several healthy lifestyle topics in their learning objectives. Some teachers expressed that while they talk about a healthy diet, physical activity, and tobacco use with their students, they need other resources because the information in the textbooks is not enough or it is outdated.

#### Enforce mandatory Physical Education (PE) requirements

In both public and private schools, PE is mandatory, and children are expected to do at least two hours each week. However, many public schools don’t have a PE teacher and in some cases the grade-level teachers (with no PE certification) are the ones who carry out PE classes. An authority representative of the General Directorate of Physical Education (DIGEF, acronym in Spanish), through the Ministry of Education explained, *“…we have more less 2800 PE teachers nationwide. That makes 30*% *coverage* [of PE classes by PE teachers at public elementary schools]. *We are extremely short, however there is a study of 1722 new job posts, but we don’t have enough money to implement them. With ‘we’ I mean the Ministry of Education, that is the institution in charge of paying our PE teachers”.* He also added that to address this gap, the DIGEF provides PE training to grade-level teachers of schools that seek their support.

#### Control foods and beverages sold in schools

It is common in Guatemalan schools to have at least one food vendor inside. School principals expressed that guidelines and regulations (e.g., hygiene, forbidden foods and beverages) can be established in the school kiosk when signing a contract, but it’s not a custom they practice frequently. Although food vendors are unaccustomed to signing contracts, they are often collaborative with the decisions made by the school staff. Some of the food vendors mentioned that they have tried to support the school principal´s decision of limiting unhealthy foods at their kiosk, but poor sales prevented them from maintaining this practice.

#### Partner with religious leaders

Families in Guatemala often attend Catholic or Protestant churches. During the home visits, 63% (n = 19) of the parents expressed that they attend religious services at least once a week, and 36% of them attend two or more services per week. Religious leaders interviewed mentioned that they not only provide spiritual advice, but also consider important providing social support and education on social issues (e.g., interpersonal relationships, family budget, health, communication). They often organize seminars or workshops with families, adults, or children. All religious leaders expressed their interest in being trained to promote healthy lifestyles in members of their congregation.

*“The Bible says that we need to take care of our body, because the body is the temple of the Holy Spirit…So here we encourage exercise.”* (Catholic religious leader)

*“We tell our brothers that obesity is not good because it causes heart problems. We even had to pray for several of them with this problem, and we always recommend them to have a strict diet and not abuse fats.”* (Protestant religious leader)

#### Engage with government programs or activities

Several government institutions are very active within their communities, and have programs and activities that promote healthier lifestyles among its residents.

Authority representatives informed that the Municipalities usually organize educational meetings where parents learn how to provide a healthy diet to their families, provide aerobic classes in buildings located throughout the community for any adult who wants to exercise, and conduct seasonal recreational activities (e.g., fairs, sports tournaments) for their residents. All these activities are free of charge.

The community health centers plan activities with public schools in their area. Authority representatives mentioned environmental sanitation, vaccination, dentistry, and delousing as their most common activities; whereas the least common are educational activities that promote a healthy diet and physical activity. Lack of health personnel often makes it difficult for health centers to meet the needs of all the schools, hence their participation with schools is not that frequent.

The Open Schools Program is a Presidential program that consists of maintaining several public schools open during weekends (Saturday and Sunday, 8:00 – 17:00 hours) to offer children and adults the opportunity to attend and participate in artistic, cultural, and sport workshops (e.g., soccer, basketball, karate, dance, circus activities), free of cost.

## Discussion

To our knowledge, this is the first formative research study in Guatemala to assess perceptions and contextual factors regarding NCD risk factors in children and their most common influences. We found that Guatemalan children, parents, and teachers have basic knowledge about the importance of a healthy diet, and can distinguish between healthy and unhealthy foods and beverages, but lack detailed information about ingredients, benefits, and consequences of a healthy diet. These findings are in accordance with previous studies conducted in the United States and Costa Rica that found that children recognized that fruit and vegetables were good for their health, but did not specify the benefits [20-22].

We found that in the case of physical activity, knowledge in children, parents, and teachers is limited. They don’t have information about the type and amounts of physical activity recommended for children, the benefits of physical activity, and long-term consequences of inactivity. Their lack of knowledge might explain why parents and teachers seem satisfied with the amount of physical activity children carry out, why they prefer them not to play actively, and why parents don’t limit the amount of time their children spend watching television. A recent study in the United Kingdom with ethnic minority children also revealed that parent´s conversations about physical activity were limited, and identified the lack of knowledge of physical activity guidelines for children as a possible barrier [23]. Lack of parental encouragement to play active games has also been noted in other LMIC [24].

Unlike nutrition and physical activity, children, parents, and teachers are well informed about the physical consequences of smoking, with the exception of secondhand smoke. Adults continually discourage smoking in children because it represents the risk factor of greatest concern. It seems that they worry most about behaviors that can become addictions (i.e., smoking, alcohol consumption) and have social consequences.

Although the results show that there are several significant factors in each level (i.e., intrapersonal, interpersonal, organizational and community) that influence NCD risk factors in children, factors at the interpersonal level were the most commonly mentioned (Figure 1). Guatemalan children (aged 10–12 years) appear to respond easily to the influence of others, particularly that of their parents and peers. The effectiveness of peers in influencing norms, attitudes, and behavior change has already been recognized [25-28]. These findings highlight the importance of not only trying to directly impact children and their environment in interventions for NCD prevention, but also those people who are close to them, such as their peers. Health interventions in other areas, such as cigarette smoking, and alcohol use have effectively used teenagers as agents for change [29-31].

We discovered many promising strategies for reducing NCD. In Guatemala City, the school is one of the most important resources in the community. First, this is the only place where children can be easily reached. Second, it is a safe environment compared to the neighborhoods where most children live. Third, it is an organized institution that can apply its own regulations (e.g., foods and beverages that can and cannot be sold in the school food kiosk) and make changes to its environment. Finally, health lessons that promote healthy lifestyles can easily be introduced in the classroom because it helps teachers meet the objectives of the National Curriculum of Education. Nevertheless, schools are limited in the influence they have in parents’ behavior and home environment. Most parents don´t interact with the school, but usually participate with other community organizations or institutions [32]. Therefore, community-based interventions should build partnerships between the schools and other community institutions. Religious leaders seem to be an important resource that can reach and influence parents that work and cannot attend school activities. Supporting previous research that faith-based organizations can promote health throughout communities [33-35]. In addition, the Municipalities and government programs have activities that promote healthy lifestyles that can be better utilized if they establish communication with public and private schools and disseminate information about the community activities available for children and their families.

The study had several key strengths and limitations. One strength of this study was the use of both methodological and participant triangulation to increase the study´s credibility. The use of different methods of data collection (i.e., FGD, semi-structured interviews, observation), and providers of evidence (i.e., children, parents, teachers) allowed for multiple perspectives as well as the ability to check findings for consistency. A limitation, which may affect the generalizability of the findings to other countries, is that it was based only in urban areas of Guatemala, and only in a low-income population. Additional work should be conducted in rural and medium- to high-income areas to see if these same patterns hold. However, we feel the study provides important information to develop a culturally appropriate and effective intervention in low-income urban children in Guatemala. Another limitation was that we were not able to explore all NCD risk factors. The harmful use of alcohol was not considered since there was no previous evidence to show that this behavior was common in school-age children. Yet, parents and teachers in all FGD raised this issue and so the moderator briefly addressed it. Further work is clearly needed on this topic. Finally, the existence and implementation of public policies that foster and protect healthy lifestyles among children were not assessed.

### Guiding principles for the intervention

A series of guiding principles were developed to assist the development of a community-based intervention for NCD prevention in Guatemalan school-age children.

● Use multi-level strategies (i.e., intrapersonal, interpersonal, organization, community) that seek to affect the most salient factors that influence NCD risk factors in children, in order to achieve a more permanent behavior change that carries until adulthood.

● Identify and invite key community members (e.g., school principals, food vendors, religious leaders) and institutions (e.g., Municipalities) that may directly or indirectly influence children’s health environment to collaborate and be part of the intervention. Political support and community partnerships have been identified as some of the core domains that affect a program´s capacity for sustainability [36].

● Consider the need teachers have for educational resources when developing the intervention. Educational resources (e.g., classroom activities) should help them meet the objectives set by the National Curriculum of Education, have clear explanations, and detailed procedures.

● Take advantage of the effectiveness of peers in influencing behavior change, and involve children as agents of change during the intervention. For example, give children the responsibility to organize fun activities (e.g., games, theatrical plays, short announcements) that promote healthy behaviors in other children.

● School food kiosks should be addressed with new and creative strategies that not only seek to provide healthier foods to children, but also maintain or improve vendor’s profits (e.g., improvement of food kiosk appearance, healthy food recipes, marketing strategies to promote healthy food to children) to enhance sustainability and prevent food vendors to discontinue these practices due to poor sales. More formative work is needed on this topic.

● Government programs or activities that promote healthy lifestyles should be identified in each community, and the communication between them and the public and private schools should be encouraged. It is recommended that school principals and authority representatives meet regularly to identify the best strategy to disseminate information of these programs to children inside the schools and their parents.

## Conclusions

The formative research study collected data about NCD risk factors in children and their most common influences. These findings should help researchers in Guatemala and similar LMIC to develop community-based interventions for NCD prevention in school-age children that are effective, feasible, and culturally acceptable.

## Competing interests

The authors declare that they have no competing interests.

## Authors’ contributions

PL drafted the manuscript, performed the analysis, and participated in the conception, design and coordination of the study. MR and BC edited the manuscript; and participated in the conception and design of the study. JG gave expert input on qualitative analysis and helped draft the manuscript. All authors read and approved the final manuscript.

## Pre-publication history

The pre-publication history for this paper can be accessed here:

http://www.biomedcentral.com/1471-2458/14/101/prepub
